# The Digital Clock and Recall can predict functional impairment in individuals with and without cognitive impairment and triage those in need of further assessment

**DOI:** 10.1002/alz.089288

**Published:** 2025-01-09

**Authors:** Marissa C Ciesla, Claudio Toro‐Serey, Ali Jannati, Joyce Rios Gomes‐Osman, Russel Banks, John Showalter, Sean Tobyne, Alvaro Pascual‐Leone

**Affiliations:** ^1^ Linus Health, Boston, MA USA; ^2^ Harvard Medical School, Boston, MA USA; ^3^ Linus Health, Waltham, MA USA; ^4^ University of Miami Miller School of Medicine, Miami, FL USA; ^5^ Michigan State University, East Landing, MI USA; ^6^ Hebrew SeniorLife, Boston, MA USA

## Abstract

**Introduction:**

Evaluation of functional dependence in activities of daily living (ADLs) and instrumental activities of daily living (iADLs) is necessary for dementia diagnosis. ADL and iADL questionnaires are typically employed, but with progressive cognitive impairment, a care partner must step in to assist with these tests, causing logistical burdens. Pre‐screening tools that triage patients in need of formal functional assessment would optimize clinical workflows. Here we evaluated whether the Linus Health Digital Clock and Recall (DCRTM), a brief automatically‐scored test of multiple cognitive domains, could concurrently predict functional impairment among individuals with and without cognitive impairment.

**Methods:**

We collected data from 922 participants in the Bio‐Hermes‐001 multi‐site study (age mean±SD = 72±6.6; 57% female; years of education mean±SD = 15±2.71; primary language English), classified as cognitively unimpaired (n = 402), mild cognitively impaired (n = 298), or probable Alzheimer’s dementia (n = 242) based on clinical consensus and neuropsychological evaluation. We split participants, each including 2000 speech and drawing DCR metrics and age, into training (80%) and test (20%) sets, ensuring equal rates of functional impairment across sets (Functional Activities Questionnaire ≥ 6; 25% rate). We performed recursive feature elimination on 10‐fold cross‐validated random forests (5 repetitions) on the training set to identify the best model and feature set. Model performance was evaluated on the test set.

**Results:**

The optimal model (500 features) classified functional impairment with 0.77 sensitivity, 0.82 specificity, 0.90 negative predictive value (NPV), 0.61 positive predictive value (PPV), 0.86 area under the receiver operating characteristic curve (AUC), and 0.80 accuracy. The most predictive features were the duration of speech during recall and the placement of clock components.

**Conclusion:**

The DCR, a brief digital cognitive assessment with automatic scoring that can be completed in primary‐care settings, can also be used to identify individuals in need of further evaluation and engagement of care partners as informants. The high NPV can reassure healthcare professionals when no further assessment of ADLs and iADLs is indicated. These results also enable identifying care partners who are likely to benefit from receiving personalized recommendations or training to assist them in caregiving tasks and alleviate caregiver burden.

